# Tripping Avoidance Lower Extremity Exoskeleton Based on Virtual Potential Field for Elderly People

**DOI:** 10.3390/s20205844

**Published:** 2020-10-15

**Authors:** Zongwei Zhang, Changle Li, Tianjiao Zheng, Hongwu Li, Sikai Zhao, Jie Zhao, Yanhe Zhu

**Affiliations:** State Key Laboratory of Robotics and Systems, Harbin Institute of Technology, Harbin 150001, China; kingwill2020@163.com (Z.Z.); hitzhengtianjiao@163.com (T.Z.); lihongwuhit@163.com (H.L.); zhaosikai1990@163.com (S.Z.); jzhao@hit.edu.cn (J.Z.)

**Keywords:** virtual potential field, tripping avoidance, exoskeleton, impedance controller

## Abstract

Tripping is a common problem that everyone faces when walking. This paper mainly focuses on a lower limb exoskeleton that can help those weak in joints to avoid tripping when negotiating stairs or stepping over obstacles. This method does not need a camera or map reconstruction to recognize the obstacles and plan paths. The exoskeleton applies an impedance controller to follow and control the pilot’s movements. A virtual potential field is proposed to help the robot regulate the pilot’s motion and avoid kicking the obstacles appearing in front of the pilot’s foot during walking. Simulation and experiments show that this method works effectively and could help the elderly and those affected by joint weakness avoid tripping when walking.

## 1. Introduction

The global trend in population aging is associated with an increased incidence of falls, resulting in morbidity, mortality, and burden to the individual, immediate family, and the social healthcare system [1,2]. Falling can be attributed to the insufficient reduction of the angular momentum and improper placement of the recovery limb [3,4]. Tripping is a significant cause of falls, especially in the elderly [5,6,7]. Trip recovery strategies mainly include either placing the obstructed leg on the ground immediately while taking a recovery step with the contra-lateral leg or elevating the obstructed leg to cross the obstacle and continue walking [8,9]. The choice of recovery response is relative to the perturbation duration and obstruction onset time in the gait cycle [3,10,11,12]. Studies on the elderly have shown that they prefer placing the obstructed leg on the ground after being perturbed but have less successful recovery rates [3,12]. The reason for lower recovery rates is associated with lower joints output [3], reduced response time [13,14], impaired speed of information processing [12], biomechanically less effective medial-stepping strategy [15], and age-related decline in anterior stepping thresholds [16]. Compared with providing adequate recovery response after tripping, avoiding obstacles before tripping by controlling foot clearance is more feasible and more accessible using a robot.

Devices that can provide power assistance at the weak or fatigue joints as well as control foot clearance would be a better choice to decrease the risk of falls during walking. An exoskeleton would be a natural choice to fulfill these demands [17]. The exoskeleton is a kind of robot that can be worn by humans and provide the pilot the additional power to walk and support loads. Until now, many types of exoskeleton have been proposed to help soldiers [18,19], workers [20,21,22,23,24], the elderly [20,25,26], and the differently-abled [27,28,29,30]. All these exoskeletons were designed to help the pilots share payloads or provide assistance during working or walking. In contrast, obstacle avoidance was a neglected aspect, which is equally important, especially during stair climbing or uneven ground walking for the elderly. Here, we propose a tripping-free exoskeleton robot to help the pilot avoid obstacles by controlling the foot clearance during climbing stairs or level walking.

## 2. Controller Design

Research on the controlling of the lower limb exoskeleton has been underway for years. A load-carrying augmentation exoskeleton can apply the sensitivity amplification method [19,31], the model-based control method [32,33], and sometimes an Electromyography (EMG)-signal-based controller [34]. Exoskeletons generally use a predefined gait trajectory to assist impaired subjects [35,36,37]. Exoskeletons can aid the elderly or weak-muscled adults with an EMG-based controller [34], a model-based controller [38,39], or simply by controllers with a predefined gait trajectory [40]. The impedance controller of the exoskeleton can control the position of the exoskeleton and control the interactive force between the human and the exoskeleton [41]. In this study, we mainly focused on avoiding tripping during the walking process. Both position and interactive forces need to be controlled. Controllers with a predefined gait trajectory may not suit different wearers, and EMG-based controllers need an additional method to deal with individually dependent signals, which makes the exoskeleton a complicated system. Thus, an impedance controller would be a better choice to meet these demands. In this section, an impedance controller of the exoskeleton is proposed to avoid tripping by controlling the distance between the human and the obstacles.

During walking, trips always occur during the swing phase [42]. The proposed method mainly focuses on the swing leg. The kinetic equation of the swing leg is:

(1) $$H\left(\theta \right)\ddot{\theta }+C\left(\theta ,\dot{\theta }\right)\dot{\theta }+G\left(\theta \right)+G_{w}\left(\theta \right)=T_{m}+T_{w},$$

where θ is the angular displacement vector in the joint space; $H\left(\theta \right),C\left(\theta ,\dot{\theta }\right)$, and G(θ) are the inertial, Coriolis, and gravitational matrices of the exoskeleton, respectively; $G_{w}\left(\theta \right)$ is the gravity matrix of the pilot; and $T_{m}$ and $T_{w}$ are torques of the exoskeleton and the pilot, respectively. We designed the control law as:

(2) $$T_{m}=\hat{H}\left(\theta \right)\alpha +\hat{C}\left(\theta ,\dot{\theta }\right)\dot{\theta }+\hat{G}\left(\theta \right)+{\hat{G}}_{w}\left(\theta \right)-J^{T}\left(\theta \right)f,$$

(3) $$\alpha =J^{-1}\left(\theta \right)\left(\beta -\dot{J}\left(\theta ,\dot{\theta }\right)\dot{\theta }\right),$$

(4) $$\beta ={\ddot{x}}_{R}+K_{M}^{-1}\left(K_{D}\Delta \dot{x}+K_{P}\Delta x+f\right),$$

where $\hat{H}\left(\theta \right),\hat{C}\left(\theta ,\dot{\theta }\right)$, $\hat{G}\left(\theta \right)$, and ${\hat{G}}_{w}\left(\theta \right)$ are the estimated matrices of $H\left(\theta \right),C\left(\theta ,\dot{\theta }\right)$, G(θ), and $G_{w}\left(\theta \right)$, respectively; *J* is the Jacobian matrix of the foot on the swing leg; *f* is the general interactive force between the pilot and the exoskeleton; $x_{R}$ is the reference trajectory from the pilot; and ${\dot{x}}_{R}$ and ${\ddot{x}}_{R}$ are the corresponding velocity and acceleration, respectively. Define $\Delta x=x_{R}-x,\Delta \dot{x}={\dot{x}}_{R}-\dot{x}$, and $\Delta \ddot{x}={\ddot{x}}_{R}-\ddot{x}$ as the error functions, and *x* is the position of the foot in Cartesian space. $K_{M},K_{D}$ and $K_{P}$ are positive definite matrices. After substituting the control law into the kinetic equation, we obtain:

(5) $$\begin{array}{c} \Delta \ddot{x}+K_{M}^{-1}\left(K_{D}\Delta \dot{x}+K_{P}\Delta x+f\right)\\ ={\hat{A}}^{-1}\left(\theta \right)\left(\Delta A\left(\theta \right)\ddot{x}+\Delta B\left(\theta ,\dot{\theta }\right)\dot{x}+\Delta D\left(\theta \right)\right), \end{array}$$

where $A=J^{-T}\left(\theta \right)H\left(\theta \right)J^{-1}\left(\theta \right)$, $\Delta A\left(\theta \right),\Delta B\left(\theta ,\dot{\theta }\right)$, and ΔD(θ) are defined as:

(6) $$\Delta A\left(\theta \right)=J^{-T}\left(\theta \right)\left(H\left(\theta \right)-\hat{H}\left(\theta \right)\right)J^{-1}\left(\theta \right),$$

(7) $$\begin{array}{c} \Delta B\left(\theta \right)=J^{-T}\left(\theta \right)\left(C\left(\dot{\theta },\dot{\theta }\right)-\hat{C}\left(\dot{\theta },\dot{\theta }\right)\right)J^{-1}\left(\theta \right)\\ -\Delta A\left(\theta \right)\dot{J}\left(\theta ,\dot{\theta }\right)J^{-1}\left(\dot{\theta }\right), \end{array}$$

(8) $$\Delta D\left(\theta \right)=J^{-T}\left(\theta \right)\left(G\left(\theta \right)+G_{w}\left(\theta \right)-\hat{G}\left(\theta \right)-{\hat{G}}_{w}\left(\theta \right)\right).$$

In the following sections, we assume that ΔA=ΔB=ΔD=0 since the uncertainties can be approximated by many adaptive methods [41,43,44]. Thus, the error kinetic equation becomes:

(9) $$K_{M}\Delta \ddot{x}+K_{D}\Delta \dot{x}+K_{P}\Delta x=-f,$$

this equation shows that if $K_{M},K_{D}$, and $K_{P}$ are properly chosen, the general interactive force *f* can be kept within a smaller amplitude. It also indicates that if *f* is modified on purpose, the exoskeleton’s walking path can be changed to the desired trajectory.

## 3. Human–Exoskeleton Interactive Force and Virtual Force

### 3.1. Human–Exoskeleton Interactive Force

Compared with traditional impedance control, the most significant difference in the impedance controller in the exoskeleton is than the reference trajectory is obtained from the human, so is unknown and needs to be estimated by the human–exoskeleton interactive force [41]. The exoskeleton–human system could be treated as a robot–environment system; thus, humans can be regarded as general environments. Many methods have been proposed to model the dynamics between a robot and environments [45,46]; the interactive force between them could be treated as a combination of inertia, damping, and spring. In this paper, a second-order dynamics equation is applied to express the interactive force between the pilot and the exoskeleton. Define the interactive force as:

(10) $$f_{I}=K_{ep}\Delta x+K_{ed}\Delta \dot{x}+K_{em}\Delta \ddot{x},$$

where $K_{ep},K_{ed}$, and $K_{em}$ are the stiffness, damping, and inertia parameters of the human–exoskeleton interactive system, respectively. After using the recursive least-squares method to estimate these parameters [47], the reference trajectory $x_{R}$ can be obtained by solving the differential Equation (11) through the numerical method [48].

(11) $$f_{I}={\hat{K}}_{ep}\Delta x+\hat{K_{ed}}\Delta \dot{x}+{\hat{K}}_{em}\Delta \ddot{x},$$

where ${\hat{K}}_{ep},{\hat{K}}_{ed}$, and ${\hat{K}}_{em}$ are the estimated values of the corresponding stiffness, damping, and inertia parameters, respectively.

### 3.2. Virtual Force

With impedance control, the interactive force is controlled indirectly by the proper adjustments of the reference trajectory [46]. In the exoskeleton robot system, the reference trajectory is estimated from the interactive force. Thus, to avoid tripping during walking, an artificial regulated force can be added to the system to adjust the reference trajectory.

**Figure 1:**
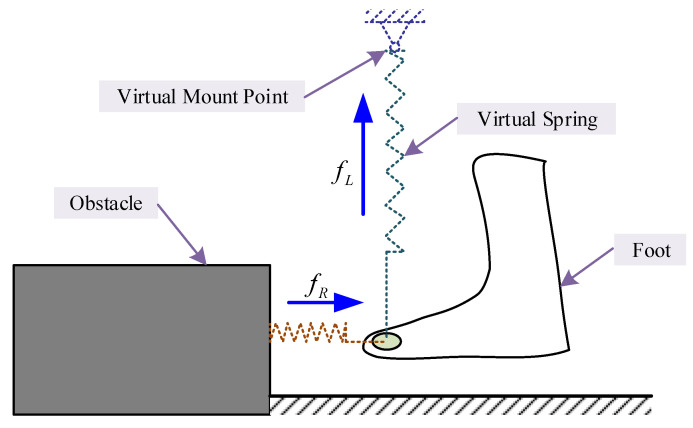
Illustration of virtual forces. $f_{L}$ and $f_{R}$ are lifting force and repulsive force generated from virtual springs, respectively. The forces only take effect on a given region.

During the walking process, falls may occur due to having less that the minimum required clearance between the foot and the obstacles [49,50,51,52]. To control the foot clearance and avoid tripping, a virtual repulsive force is used to help the exoskeleton robot adjust the foot clearance. The repulsive force is designed as a non-negative continuous and differentiable function [53] that reaches infinity as the foot approaches the surface of the obstacles. To avoid undesired perturbation beyond the vicinity of the obstacle, the effective scope of the repulsive force should be limited in a given region. Define the repulsive force as:

(12) $$f_{R}=\left\{\begin{array}{cc} \kappa_{R}\left(\frac{1}{\rho_{R}}-\frac{1}{\rho_{0}}\right) & \rho_{R}\leq \rho_{0}\\ 0 & \rho_{R}>\rho_{0} \end{array}\right.,$$

where $\kappa_{R}$ is a constant gain, $\rho_{R}$ is the clearance to the surface of the obstacle, and $\rho_{0}$ is the predefined scope of the repulsive force to work. When the foot is going to hit the obstacle, the repulsive force will take effect. It seems like the foot is touching a virtually flexible wall with no friction and can deform a little by compressive force from the pilot’s foot. Since humans use dynamic balance strategies during walking [54,55], a lifting force from the foot will help the pilot step over the stair as quickly as possible. Assume there is a virtual spring over the foot lifting the foot during the clearance controlling stage and the lifting force is related to the distance from the foot to the virtual spring mount point (Figure 1). Define the lifting force as:

(13) $$f_{L}=S\kappa_{L}\left(\rho_{S}-\rho_{L}\right),$$

where $\kappa_{L}$ is a constant gain, $\rho_{L}$ is the distance from the foot to the ground surface, $\rho_{S}$ is the position of the virtual mount point, and $S=1/(1+e^{-t})$ is a transition function used to avoid sudden change in the lifting force, where *t* is the transition time. After the exoskeleton helps the pilot lift their foot and avoid scuffing between the foot and the obstacle, the distance between the foot and obstacle increases dramatically, and Equation (12) no longer satisfies the requirements. Thus, a blending function (the *S* transition function is used in this simulation) is needed to bring the large virtual forces to zeros to avoid instability of the system. The general interactive force can be expressed as:

(14) $$f=f_{I}+f_{R}+f_{L}.$$

Thus, the new reference trajectory obtaining equation can be rewritten as:

(15) $$f={\hat{K}}_{ep}\Delta x+{\hat{K}}_{ed}\Delta \dot{x}+{\hat{K}}_{em}\Delta \ddot{x}.$$

## 4. Simulation

A lower-limb exoskeleton robot with three degrees of freedom on each leg was applied to execute the trip avoiding simulations. The exoskeleton joints were placed on the hip joint of the sagittal and frontal plane and knee joint of the sagittal plane. The simulation concentrated on the sagittal plane since the main active joints were placed in that plane, and the walking trajectory was in that plane too. A range sensor was installed on the sole to measure the distance between the foot and the obstacle. If the foot steps into the repulsive force influence region (tripping area), a virtual repulsive force will be generated to correct the robot’s current trajectory to maintain a safe clearance. To better illustrate the method, we used a doorsill-like obstacle as an example to test the controller’s effectiveness. The obstacle used in the simulation was 170 mm tall and 100 mm wide.

**Figure 2:**
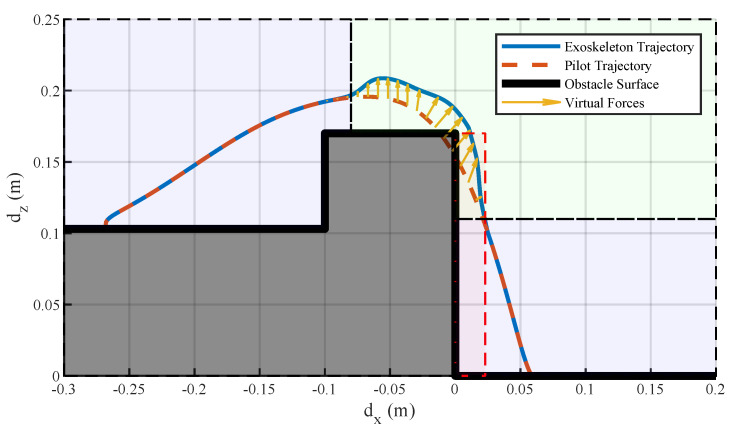
Trajectories of the foot during stepping over the obstacle. The pilot trajectory is a given path that would cause a fall, and the exoskeleton trajectory is the successful obstacle avoidance path after the virtual forces take effect. The short lines with arrows are the resultant forces of the repulsive force and the lifting force generated according to the foot clearance. The trajectory tracking area indicates the exoskeleton following the pilot’s movement. The obstacle avoidance area indicates the exoskeleton regulating the pilot’s movement, and the tripping area indicates a high probability of tripping if no actions are taken during stepping over obstacles.

Simulations were conducted with and without using the virtual potential field. Figure 2 shows the corresponding trajectories with and without using the virtual forces to step over the doorsill. The virtual forces were triggered by the clearance between the foot and the doorsill. When the foot clearance was under 20 mm, the probability of the pilot’s foot scuffing the surface of the obstacle would be high, and falling would happen. In these situations, the exoskeleton had the responsibility to correct the running trajectory. When the foot stepped into the assumed tripping area, a repulsive force was generated to push the foot away from the obstacle. Meanwhile, a lifting force was generated to help the pilot to easily lift their leg. After the pilot lifted their leg high enough and avoided the obstacle, the repulsive force would vanish, and only the lifting force was held to protect the pilot from kicking the top surface of the obstacle. The virtual forces shown in the figure are the resultant forces of the repulsive force and lifting force. The length of the lines indicates the magnitude of the resultant forces, and the direction of the lines is the direction of the resultant acting force.

**Figure 3:**
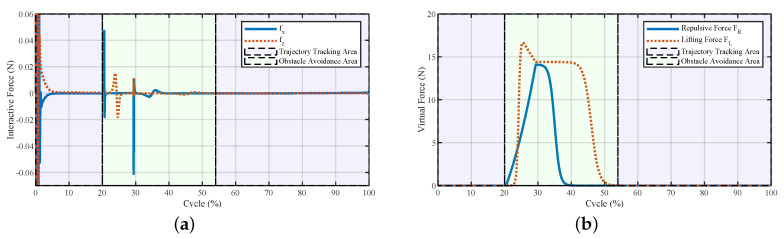
Interactive forces and the virtual forces of the system generated during stepping over the obstacle. (**a**) Human–exoskeleton interactive forces and (**b**) repulsive force and lifting force.

The interactive forces and the virtual forces generated during stepping over the obstacle are illustrated in Figure 3. At the beginning of the obstacle avoidance area, both the repulsive and lifting forces acted on the exoskeleton to help the pilot maintain the foot clearance and lift their foot easily. After 40% of the swing cycle, the repulsive force vanished, and the lifting force was still maintained to help the pilot keep the distance between the foot and the top surface of the obstacle. The interactive forces $f_{x}$ and $f_{z}$ showed that the total interactive forces were relatively low, which means that from Equation (14), the interactive force $F_{I}$ between the exoskeleton and the pilot would be more significant in the obstacle avoidance area when changing the current walking trajectory.

**Figure 4:**
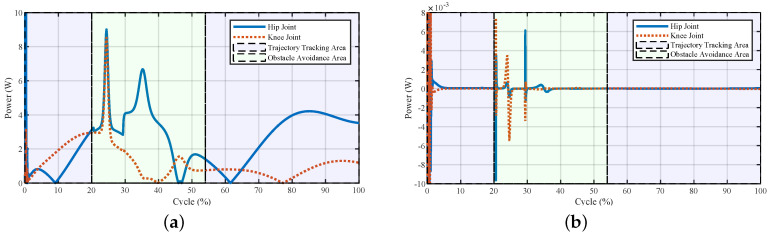
Joint powers of the system during stepping over the obstacle. (**a**) Powers of the exoskeleton and (**b**) powers of the interaction. The interaction powers reflect how much power was output by the pilot to drive the exoskeleton or power the robot outputted to help the pilot.

**Figure 5:**
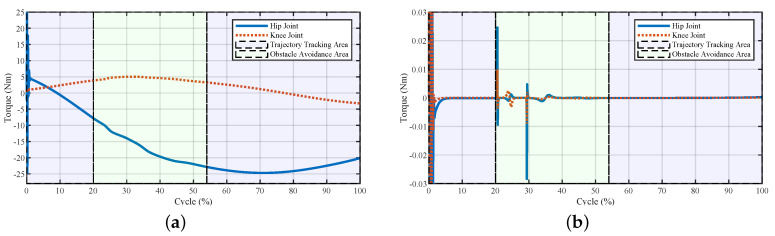
Joint powers of the system during stepping over the obstacle. (**a**) Torques of the exoskeleton and (**b**) torques of the interaction.

The powers and torques of the exoskeleton and the human–exoskeleton interaction are illustrated in Figure 4 and Figure 5. The interaction powers and torques reflect how much force was required by the pilot output to drive the exoskeleton or the forces output by the exoskeleton to help the pilot. We observed that the joint powers and torques from the pilot were low, which meant that the pilot only provided little force to swing the leg to avoid the obstacle. The powers of the exoskeleton showed that after the virtual forces were added to the system, the power first increased at a relatively high rate (about 25% of the cycle); this was the appearance of the larger repulsive force and lifting force (Figure 3). After that, with the increase in the foot height, the exoskeleton’s power decreased. After 30% of the cycle, there were fluctuations both in the powers and torques of the exoskeleton and pilot; this was the pulse of the repulsive force and lifting force at the transition point. After that point, the repulsive force decreased to zero, and the lifting force would hold for a while to support the pilot and then decreased. The powers and torques from the pilot reflected this trend. The exoskeleton torques showed that the fluctuations caused by the virtual forces were relatively small, and the fluctuations could be treated as a dynamic tracking problem. After the virtual forces were added to the system, the reference trajectory changed and the robot needed to track the new trajectory. From the controller’s structure, if the parameters of the closed-loop system equation were carefully chosen and the functions of virtual force were continuous, the system would be stable.

## 5. Experiment

**Figure 6:**
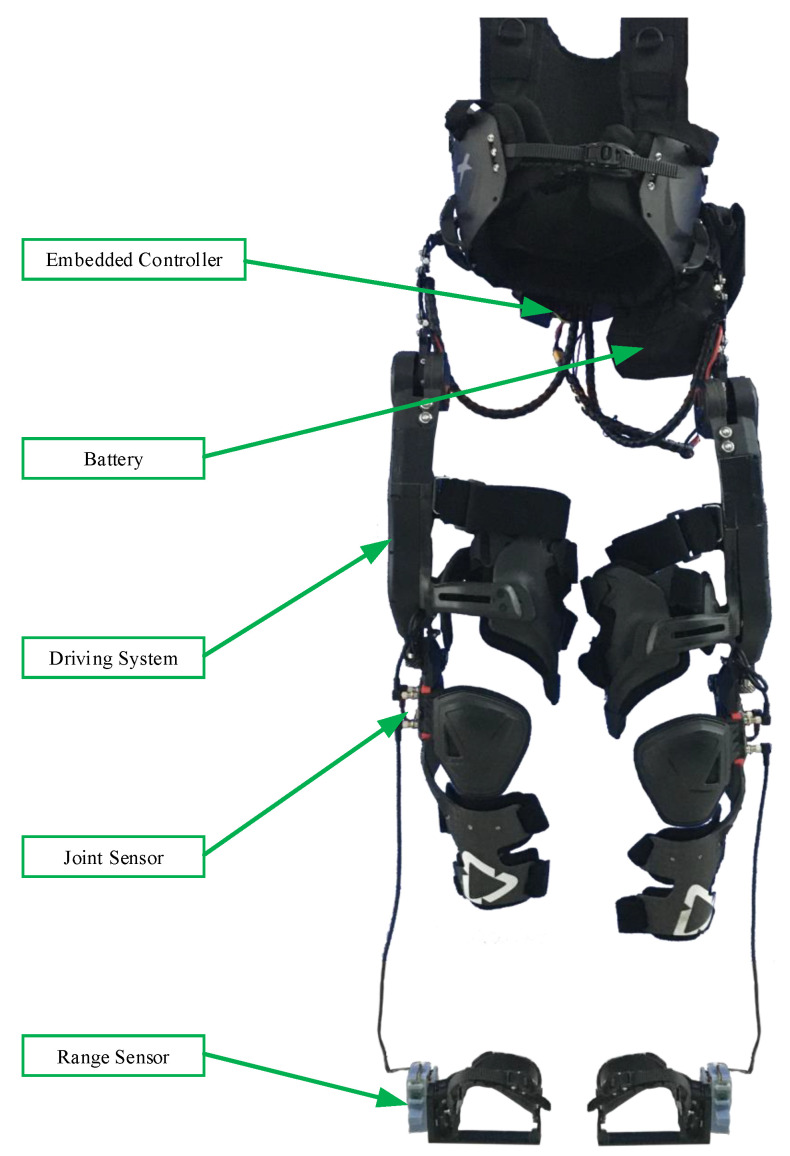
Exoskeleton prototype. The exoskeleton robot had active hip and knee joints on the sagittal plane and a negative hip joint on the frontal plane.

Experiments using the proposed method were conducted on a lower limb exoskeleton (Figure 6) with three joints (active hip and knee joints on the sagittal plane and negative hip joint on the frontal plane). Force sensors (JLBS-MD, Zhongwan Sensor, Anhui, China) were installed on each powered joint to measure the interactive forces between the exoskeleton and the pilot. The exoskeleton had two time-of-flight ranging sensors (ST™VL53L3CX with a range up to 300 cm and accuracy ±2.5%) on each foot to measure the distance between the foot and the obstacles and the ground, separately. A pilot wore the exoskeleton walked on the ground with different obstacles to test the effectiveness of the method, and the trajectories of the exoskeleton robot to avoid obstacles with different shapes are recorded and illustrated.

### 5.1. Step Over a Door Threshold

**Figure 7:**
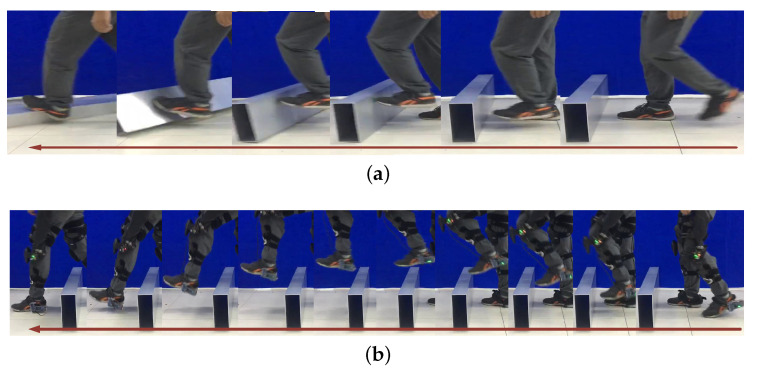
Experiment with stepping over a door threshold; the arrow indicates the walking direction of the pilot. (**a**) Illustration of tripping during stepping over a door threshold. (**b**) Experiment involving stepping over a door threshold with the help of the exoskeleton.

To illustrate the tripping process during stepping over a door threshold, a tripping example is shown in Figure 7a. In the example, the door threshold was not fixed; if a person kicked the door threshold, the door threshold would move, which indicated that the pilot would be tripped by the door threshold. During the walking process, if the pilot could not swing their leg high enough, which usually occurs for those with weak joints, or if someone is not aware of the obstacle, their foot would kick aside the door threshold. In the real world, the door threshold is fixed and if the force from the pilot did not disappear quickly, the pilot would be tripped, so some measures need to be taken to avoid this danger.

**Figure 8:**
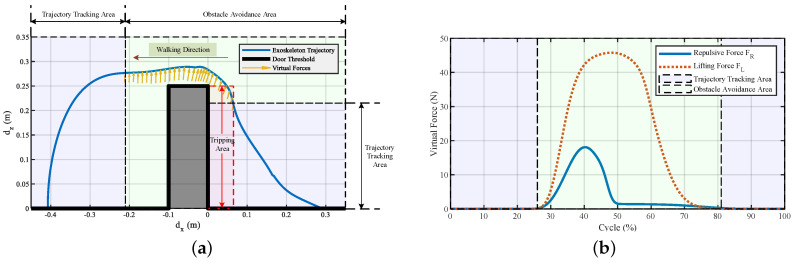
Trajectory and virtual forces of the exoskeleton to step over a door threshold. (**a**) Trajectory of the exoskeleton, the arrows denote the generated resultant force of repulsive and lifting forces. The dotted red rectangle indicates the danger area that would cause tripping if a person could not swing their leg high enough. (**b**) Repulsive and lifting forces generated during stepping over a door threshold. $F_{R}$ is the repulsive force used to help the pilot avoid kicking the door threshold, $F_{L}$ is the force used to help the pilot lift their leg. The repulsive force and the lifting force worked in the green area.

Figure 7b shows the experiment that involves stepping over a door threshold with the exoskeleton’s help. The trajectory to step over the door threshold (250 mm in height and 100 mm in width) is shown in Figure 8a; only the swing phase in the sagittal plane is shown since the motion and active joints were in that plane. The figure shows that after the pilot lifts their leg 0.21 m high, a repulsive force and a lifting force appear to help them avoid kicking the door threshold and lift their leg. The repulsive force was active when the distance between the foot and the door threshold was below 6 cm, and Figure 8b depicts the corresponding repulsive and lifting forces. The dotted red rectangle in Figure 8a is the assumed danger area that would cause tripping if the person could not swing their leg high enough. The yellow arrows are the virtual forces generated during the method working process; the length and direction of the arrows indicate the magnitude and direction of the virtual forces, respectively. The repulsive force aimed to push the foot away from the door threshold to avoid kicking it, and the lifting force was used to help the pilot swing their leg high enough. At about 26–50% of the cycle, the repulsive and lifting forces worked together to help the pilot step over the obstacle. From 50–81% of the cycle, the lifting force became the major force to help the pilot swing their leg.

**Figure 9:**
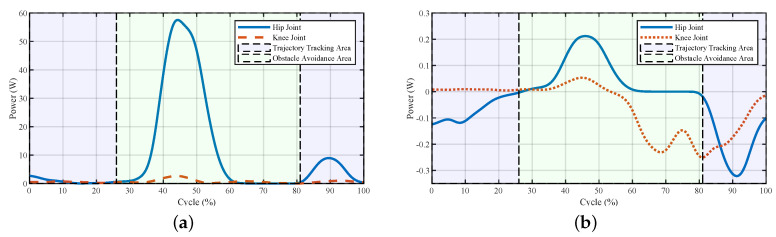
Joint powers of the system to avoid tripping during stepping over the door threshold. (**a**) Powers of the exoskeleton and (**b**) powers of the interaction. The exoskeleton’s more considerable power reflected that the virtual forces took effects, and the pilot’s corresponding powers showed that there were confronted forces between the robot and the pilot.

**Figure 10:**
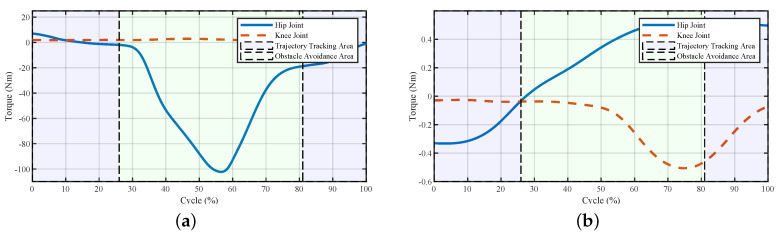
Joint torques of the system used to avoid tripping during stepping over a door threshold. Torques of the (**a**) exoskeleton and (**b**) interaction.

The corresponding powers and torques of the exoskeleton and the human–exoskeleton interaction are shown in Figure 9 and Figure 10, respectively. Since the pilot’s torques and powers were hard to measure, we used the human–exoskeleton interaction torques and powers instead. The interaction torques and powers reflected how much force was required by the pilot output to drive the exoskeleton or the force output by the robot to help the pilot, which illustrated the power and torque of the pilot.

At the beginning of the tripping avoidance (26% of the cycle), the powers of the exoskeleton both on the hip and knee joint were increased to drag the foot away from the door threshold and lift the leg. After about 35% of the cycle, the power of the hip joint increased significantly. The reason for this was the increase in the lifting force and the hip joint angle to step over the door threshold. The hip joint needed to balance the gravitational force of both the exoskeleton and the pilot. At about 45–81% of the cycle, the powers of both the hip and knee joints started decreasing due to the decreases in lifting and repulsive forces. The torques of the exoskeleton reflected the same trend as the powers. The powers of the interaction showed the interactive process between the pilot and the exoskeleton. The larger powers among 35–60% indicated that there were forces between them, and the exoskeleton tried to change the pilot’s movement to avoid tripping. The interaction torques showed that during the obstacle avoidance period, the torques from the pilot increased until 75% of the cycle and then decreased to a small value at the knee joint. After about 81% of the cycle, with the disappearing of repulsive and lifting forces, the interaction powers increased to help the pilot swing the leg to put their foot on the ground.

### 5.2. Step Over Obstacles

**Figure 11:**
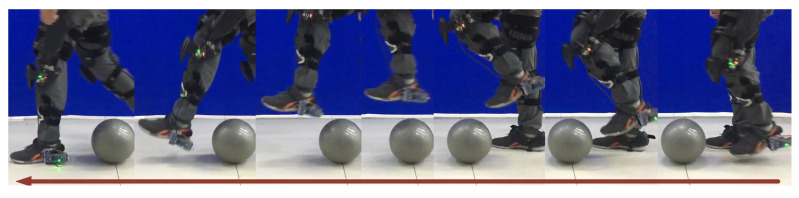
Stepping over an obstacle during walking. The arrow indicates the walking direction of the pilot.

**Figure 12:**
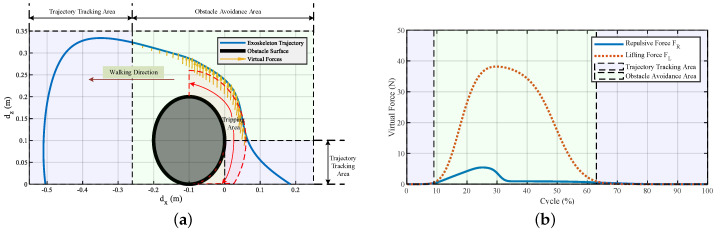
Trajectory and virtual forces of the exoskeleton system during stepping over a ball-like obstacle. (**a**) Trajectory of the exoskeleton. The dotted red area is the dangerous area that might cause scuffing if the swing leg could not be controlled. (**b**) Repulsive and lifting forces generated during stepping over a ball-like obstacle. $F_{R}$ and $F_{L}$ are the forces used to help the pilot avoid kicking the obstacle and lift the swing leg, respectively.

In daily life, everyone needs to step over obstacles when walking. The obstacles have different kinds of shapes, and ball-like obstacles may be the most common. We conducted an experiment on stepping over a ball-like obstacle to demonstrate the working process of the proposed method (Figure 11). The trajectory of the exoskeleton to overcome a ball-like obstacle is shown in Figure 12a. The virtual force was triggered in the dotted red area, and the virtual repulsive force and lifting force are illustrated in Figure 12b. The virtual forces took effect at about 9% of the cycle and ended at about 63% of the cycle. The repulsive force worked at about 9–35% to maintain the distance between the foot and the obstacle to avoid scuffing between the foot and the obstacle; then, it decreased to zero. At the same time, the lifting force worked at about 9–63% of the cycle to help the pilot lift their swing leg to step over the obstacle.

**Figure 13:**
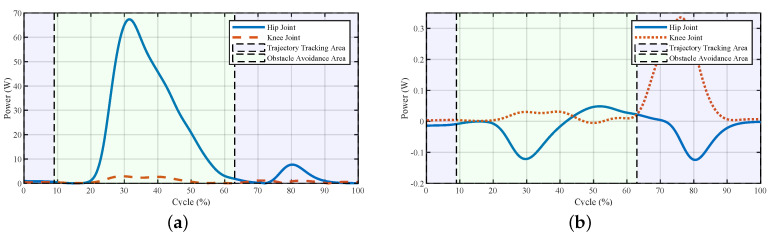
Joint powers of the system during stepping over a ball-like obstacle. (**a**) Powers of the exoskeleton and (**b**) powers of the interaction. The larger powers of the exoskeleton meant that the exoskeleton tried to change the current movement, and larger powers from the pilot indicated that the pilot tried to dominate the movements.

**Figure 14:**
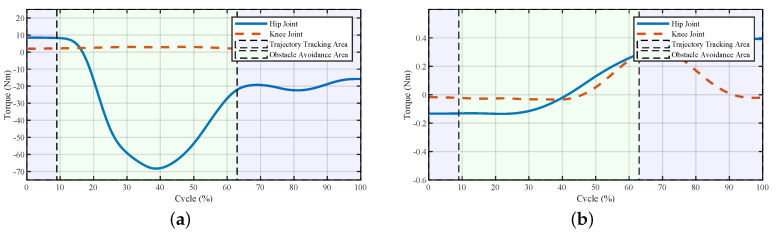
Joint torques of the system during stepping over a ball-like obstacle. (**a**) Torques of the exoskeleton and (**b**) torques of the interaction.

The powers and torques of the exoskeleton and the interaction between the pilot and the exoskeleton are shown in Figure 13 and Figure 14, respectively. At about 9–32% of the cycle, the power of the interaction in the hip joint increased, reflecting the leading force from the exoskeleton to change the current positions of the pilot. The forces between the pilot and the exoskeleton were small since the pilot adjusted their movement to catch up with the pace of the exoskeleton. In the second half of the trajectory, the interaction torques on both joints increased a certain extent, which meant the pilot tried to lift their leg. The reason for this was that after 63% of the cycle, the lifting force decreased, and the pilot felt less of a lifting force from the robot, which led the pilot to lift their leg subconsciously. The powers and torques from 63–75% of the cycle in Figure 13b and Figure 14b reflect this phenomenon. After the robot helped the pilot surmount the obstacle, the pilot controlled the exoskeleton to finish the current step.

## 6. Discussion and Future Works

This paper presented an impedance control exoskeleton robot to avoid obstacles by using a virtual potential field. Simulations and experiments were conducted to validate the effectiveness of the proposed method. The simulations and experiments showed that the proposed method could help the pilot avoid tripping, but the differences between them should be analyzed. The pilot’s powers in the simulation were small, whereas in the experiments, they were larger both in the obstacle avoidance and trajectory tracking areas. The reason for this was that in the experiments, the interactive properties between the pilot and the exoskeleton were not constant; they varied between different pilots or even different experimental times. The impedance controller’s design goal was to let the pilot use little force to swing their leg, and the ideal interactive powers of the pilot were zero, which was reflected in the simulation. In the experiments, the pilot’s interactive powers were not zero, but compared with the exoskeleton powers, they were small enough to satisfy the design. The pilot’s intention in the experiments was uncertain, which could lead to both cooperation and opposition between the exoskeleton and the pilot. The pilot would change their motion based on the current walking status, not only due to the forces from the exoskeleton.

Considering the differences between individuals, the triggering of the virtual forces would differ. In our experiments, the threshold values were obtained by experiments. In the future, a machine learning method would be a better choice to obtain these values through fewer experiments. In our experiments, we only considered the swing leg, but the human is a whole; the whole body stability should be considered. Since the pilot walked at a relatively slow speed (0–1 m/s) in our experiments, the influence of the swing leg on the whole stability was not that obvious, and the pilot had enough time to adjust their balance. If the method were to be applied in a factory working exoskeleton or military operation exoskeleton that emphasized speed, the whole body stability should be considered. The opposing forces also affect whole body stability; the large confronting forces indicated that the exoskeleton tried to change the motion of the pilot or vice versa. Under an extreme case, if the pilot did not change their motion during the tripping avoidance process and intentionally output large forces to maintain the current walking trajectory, the confronting forces would be high, and the pilot would fail to keep the whole body stable, which means the exoskeleton would need to help the pilot control the stability by giving up current actions or changing to other walking modes. In the future, a tripping-free exoskeleton to help both persons with or without disabilities to walk on rough roads as they wish without staring at the complex terrain would be an interesting area of research.
